# Supplementary material for: EnzML: multi-label prediction of enzyme classes using InterPro signatures
Source: BMC Bioinformatics. 2012 Apr 25;13:61. doi: 10.1186/1471-2105-13-61 (PMC3483700; doi:10.1186/1471-2105-13-61)
Supplement: Addtional file 5 — The Java code to format the data files, evaluate and predict. The file enzml_java_code.tar.gz contains the Java code used to format database data to ARFF and XML formats, to execute cross and train-test (jackknife) evaluations and to record evaluation results to database. More information is included in the readme.txt file and the Javadoc files. The code can be used with a MySQL database. To use a different database software, other JDBC drivers might be required. [file 1471-2105-13-61-S5.gz › java_code/enzml2011/doc/uk/ac/ed/inf/enzml/mulan/learn/database/MulanDbCreator.html]

MulanDbCreator


---


|  |  |  |
| --- | --- | --- |
| **Overview** \| **Package** \| **Class** \| **Use** \| **Tree** \| **Deprecated** \| **Index** \| **Help** | |  |
| PREV CLASS   **NEXT CLASS** | **FRAMES**    **NO FRAMES**     **All Classes** |  |
| SUMMARY: NESTED \| FIELD \| CONSTR \| METHOD | DETAIL: FIELD \| CONSTR \| METHOD |  |


---


## uk.ac.ed.inf.enzml.mulan.learn.database Class MulanDbCreator

```
java.lang.Object
  uk.ac.ed.inf.utils.database.DbManaged
      uk.ac.ed.inf.utils.database.DbCreator
          uk.ac.ed.inf.enzml.mulan.learn.database.MulanDbCreator
```

---

``` public class MulanDbCreator extends uk.ac.ed.inf.utils.database.DbCreator ```

Creates the table to store Mulan machine learning results. The database
(called 'mulan') must already have been created and a user with
reading/writing rights must be available.

**Version:**
:   9 Jun 2010

**Author:**
:   Luna De Ferrari luna.deferrari-at-ed.ac.uk

---

| **Field Summary** | |
| --- | --- |
| `static java.lang.String` | `MULAN_DATABASE_NAME` |


**Constructor Summary**

`MulanDbCreator(uk.ac.ed.inf.utils.database.DbManager dbManager)`


| **Method Summary** | |
| --- | --- |
| `boolean` | `createExperimentsTable()` |
| `void` | `createTables()` |

**Methods inherited from class uk.ac.ed.inf.utils.database.DbCreator**

`dropTable, getSqlToDropTable`

**Methods inherited from class uk.ac.ed.inf.utils.database.DbManaged**

`getDbConnection, getDbManager`

**Methods inherited from class java.lang.Object**

`equals, getClass, hashCode, notify, notifyAll, toString, wait, wait, wait`

**Field Detail**

### MULAN\_DATABASE\_NAME

```
public static final java.lang.String MULAN_DATABASE_NAME
```

**See Also:**: Constant Field Values


**Constructor Detail**

### MulanDbCreator

```
public MulanDbCreator(uk.ac.ed.inf.utils.database.DbManager dbManager)
```


**Method Detail**

### createExperimentsTable

```
public boolean createExperimentsTable()
```

---


### createTables

```
public void createTables()
```


---


|  |  |  |
| --- | --- | --- |
| **Overview** \| **Package** \| **Class** \| **Use** \| **Tree** \| **Deprecated** \| **Index** \| **Help** | |  |
| PREV CLASS   **NEXT CLASS** | **FRAMES**    **NO FRAMES**     **All Classes** |  |
| SUMMARY: NESTED \| FIELD \| CONSTR \| METHOD | DETAIL: FIELD \| CONSTR \| METHOD |  |


---
